# Image Segmentation of Fiducial Marks with Complex Backgrounds Based on the mARU-Net

**DOI:** 10.3390/s23239347

**Published:** 2023-11-23

**Authors:** Xuewei Zhang, Jichun Wang, Yang Wang, Yanwu Feng, Shufeng Tang

**Affiliations:** 1 State Key Laboratory of Fluid Power and Mechatronic Systems, Zhejiang University, Hangzhou 310058, China; 2 Inner Mongolia Autonomous Region Special Service Intelligent Robot Key Laboratory, Inner Mongolia University of Technology, Hohhot 010051, China

**Keywords:** fiducial mark, U-Net, residual block, CBAM, mARU-Net

## Abstract

Circuits on different layers in a printed circuit board (PCB) must be aligned according to high-precision fiducial mark images during exposure processing. However, processing quality depends on the detection accuracy of fiducial marks. Precise segmentation of fiducial marks from images can significantly improve detection accuracy. Due to the complex background of PCB images, there are significant challenges in the segmentation and detection of fiducial mark images. In this paper, the mARU-Net is proposed for the image segmentation of fiducial marks with complex backgrounds to improve detection accuracy. Compared with some typical segmentation methods in customized datasets of fiducial marks, the mARU-Net demonstrates good segmentation accuracy. Experimental research shows that, compared with the original U-Net, the segmentation accuracy of the mARU-Net is improved by 3.015%, while the number of parameters and training times are not increased significantly. Furthermore, the centroid method is used to detect circles in segmentation results, and the deviation is kept within 30 microns, with higher detection efficiency. The detection accuracy of fiducial mark images meets the accuracy requirements of PCB production.

## 1. Introduction

Printed circuit boards (PCBs) have a multilayer composite structure, are key components in electrical connections between electronic components, and are widely used in electronic products. Circuits on each layer are usually processed by a mask-less, double-sided, multi-layer exposure method [1,2]. Connections are required between each layer of circuitry and there are usually some fiducial marks on the board that serve as alignments between them. Consequently, the shape and position of these marks is very important for PCB quality [3]. With the rapid development of machine vision technology, optical imaging approaches have gradually come to play essential roles in fiducial mark detection. However, it remains a challenge to accurately detect fiducial marks due to the complex backgrounds of PCB images taken in production, which decreases the alignment accuracy of circuits between layers.

Generally speaking, it is preferable to first separate the target regions of fiducial marks from complex backgrounds, which can minimize interference from backgrounds. Traditional image-segmentation methods are based mainly on discontinuity and similarity [4], while earlier approaches used to image segmentation mainly included threshold-based methods, edge-based methods, region-based methods, methods based on graph theory, methods based on energy functionals, and others [5,6,7]. However, these traditional segmentation methods were mainly suitable for scenes where image backgrounds ere simple and uniform.

As a result, to accurately inspect fiducial marks, deep learning methods with excellent performances have been considered in the image segmentation field. Deep learning-based image segmentation technology has made significant progress [8,9]. In recent decades, hundreds of deep learning-based methods have emerged, with semantic image segmentation one of the fastest growing method areas. Traditional semantic segmentation methods pay more attention to the multi-category information of pixels rather than to edge details in the target foreground [10,11,12]. However, the segmentation of fiducial marks is not a multi-classification task but must accurately distinguish the target foreground and complex background. There are significant challenges in the segmentation and detection of fiducial mark images from complex background images. To conquer this problem, in this paper, we proposed a mARU-Net image-segmentation method based on a simplified U-Net for fiducial mark inspection.

The contributions of this paper are as follows:

- We propose a two-stage fiducial mark detection method for images with complex backgrounds. Firstly, pixel level regions of interest are extracted. Secondly, target parameters are determined using centroid detection methods.
- The mARU-Net model is proposed for the image segmentation of fiducial marks with complex backgrounds to improve detection accuracy. This model has the advantages of fewer images, fast training speeds, and low hardware requirements.
- In the proposed model, residual blocks are used to replace convolutional blocks in traditional U-Net, which improves the accuracy by 1.755%; the attention model CBAM is embedded into residual blocks to further improve image-segmentation performance, which increased the accuracy by 1.621%.

The rest of this paper is organized as follows. Section 2 summarizes classical inspection approaches for PCB components. In Section 3, our image-segmentation method is described in detail. Relevant experiments are presented in Section 4. Discussions on our study limitations and quantitative results are outlined in Section 5. Conclusions are provided in Section 6.

## 2. Related Work

In this section, we review several existing, representative vision-based detection methods for all kinds of PCB components. These can be divided into two groups: conventional image-processing-based and deep-learning-based approaches.

Conventional methods are based on three groups of image processing algorithms, (i) template-matching, (ii) image histograms, and (iii) pixel clustering.

Template-matching-based methods detect target objects by comparing similarities between the local window in the tested image and the predefined template image. Crispin et al. [13] proposed a template matching localization method for PCB component inspection that used gray-model fitting to generate common templates for a set of components, and considers normalized cross-correlation (NCC) as a measure of similarity. Hao et al. [14] selected an edge template from the X-ray image of a PCB through object extraction and threshold segmentation, and used the direction vector of the edge template as prior knowledge. However, these kinds of approaches are not robust enough due to their high sensitivity to small differences in the texture of target structures. They are also inefficient in term of large computational costs of a global traversal search.

Histogram-based methods identify regions of interest (ROIs) in PCB images by means of a comparison between pixel gray values and a calculated threshold. Sanguannam et al. [15] presented a novel, adaptive threshold segmentation approach in the field of ball grid array (BGA) quality detection, which used mean and standard deviation values of pixel gray values to calculate the threshold. Abdelhameed et al. [16] and Wu et al. [17] combined the Ostu method and discrete cosine transform (DCT) to extract solder joints. Gao et al. [18] applied an adaptive threshold method combined with modified $\left(\varepsilon ,\delta \right)$-component segmentation to extract grayscale images from solder balls. However, histogram-based methods ignore the spatial relevance of ROIs and are easily interfered with by other highlighted components.

Pixel clustering-based methods divide images into different regions based on color similarity. Zeng et al. [19] first used an automatic thresholding approach to detect specular areas which contained solder joints, and then developed a novel approach based on a connection graph and the segmented grayscale image to classify all recognized areas as several clusters. Zeng et al. [20] proposed a novel lighting scheme of three layers of ring-shaped LEDs, which yielded some customized color-distribution patterns for solder joints. Then, all solder joints of a component were clustered based on their color-distribution directions. However, these approaches depend on prior image segmentation steps and can with difficultly, meet time efficiency requirements.

Although the above conventional image processing-based methods have been used with much success in recent decades, they face various restricting factors for specific applications. With the rapid development of deep-learning technology, convolutional neural network-based (CNN-based) methods can greatly improve the accuracy of component detection. Specifically, these methods mainly include: object detection-based methods, semantic segmentation-based methods, refactor-based anomaly detection methods, etc.

Object detection-based methods need to extract ROIs from an input image and determine the category and location description of each target object. Hu et al. [21] proposed a new PCB-defect detection network based on Faster RCNN [22], which used ResNet50 with feature pyramid networks (FPN) as the backbone on the stage of feature extraction, and merged GARPN to predict more accurate anchors. Dai et al. [23] introduced a one-stage and end-to-end detection network named YOLO [24] to their localization problem of solder joints. Adibhatla et al. [25] selected a new model termed YOLO-v5 to detect defects in PCBs thanks to the model’s efficiency and accuracy. However, these approaches only provided detection results for rectangular boxes. This meant they still could not meet the accuracy requirement of pixel-level detection and localization in some industrial areas. Furthermore, a prerequisite exists for the successful application of these methods, which is to construct a dataset containing thousands of labeled samples.

Semantic segmentation is one of fastest growing areas in computer vision, with a variety of applications. Compared to object detection-based methods, semantic segmentation provides more reliable detection results than object detection because it builds a precise contour of targets. Xia et al. [26] used a region-based, fully convolutional network (R-FCN) [27] to identify minor defects on a PCB and merged and embedded focal loss and high-definition feature extraction algorithms to improve recognition rates. Tsai et al. [28] proposed a deep neural network regressor for fast and accurate image alignment when applied to PCB positioning. For PCB positioning tasks, the training image blocks and their ground response are synthesized and created for use in deep neural network models. This method only marks template patches in one PCB image, minimizing human intervention and eliminating tedious labeling work on training images. The semantic segmentation-based method is a good choice for precise positioning in a PCB component, but it still needs to overcome the restrictions of a large-scale model and thousands of training samples.

In addition, the application of deep learning to defect detection is extensive when adapting to industrial scenarios, while refactor-based anomaly detection methods have been widely studied [29,30]. Fei Liu et al. [31] proposed a new method which utilized progressive reconstruction and hierarchical feature fusion to detect packaging chips. Bing Tu et al. [32] established a new hyperspectral anomaly detection method based on reconstruction fusion, which improved robust detection performances. Yi-Xuan Xu et al. [33] constructed a novel reconstruction-based anomaly detector based only on a completely random forest, which achieved consistently better detection accuracy. Reconstruction-based anomaly detectors have many advantages, but their performances are limited by the fact that the neural network autoencoder requires a large training set and high computational costs to achieve high accuracy.

U-Net has generated significant interest in semantic segmentation image methods in many fields, including PBC detection. The U-Net model is widely used in medical image analysis. Sharma et al. [34] used a U-Net model based on deep learning which was created for small image sizes so that local segmentation features could be enhanced and extracted efficiently. They generated a technique to segment gastrointestinal organs to assist radiation oncologists treat cancer patients more accurately. Kälber et al. [35] applied U-Net [36] to binary image segmentation and used clustering algorithms to selectively group pixel coordinate labels. They used DeepPCB, a binary PCB dataset for defect detection, to achieve high segmentation performances in PCB image segmentation. Furthermore, inspired by deep residual learning and U-Net, some researchers [37,38] built a deep residual U-Net, a framework that took advantage of both deep residual learning and U-Net architecture strengths. The architecture not only eased training but also made it possible to design simple neural networks with much fewer parameters.

This research has shown that the application of deep learning has significantly improved the accuracy and efficiency of PCB component detection. In this article, we explore the use of deep-learning techniques to tackle fiducial mark image detection in PCB manufacturing. Significantly, the U-Net model demonstrated considerable advantages in the accurate segmentation of images with complex backgrounds, and required extremely small demands on the number of training samples. As a result, this paper uses the simplified U-Net as its basis and establishes the mARU-Net framework by introducing the residual learning and convolutional block attention module to segment fiducial mark images. The following section provides the architectural details of the proposed model.

## 3. Methods

In this paper, we propose a framework for fiducial mark inspection. Given an image, the task of fiducial mark inspection is to determine the center and radius of each circular fiducial mark. In order to minimize the interference of a complex background, we inspected fiducial marks in two steps: Firstly, to obtain a sufficiently fine target area, a simplified but effective semantic segmentation network was constructed based on U-Net. Secondly, a simple centroid method was utilized to compute the center and the radius.

The specific detailed description is as follows. Firstly, a customized fiducial mark image dataset was created by independent labeling and data augmentation. Secondly, by streamlining U-Net, the network structure of mU-Net was established, which ran on limited GPU hardware. Subsequently, to further optimize the network structure, the mResU-Net was established by replacing the original convolutional structure with a residual structure on the mU-Net path. To further improve segmentation accuracy, the final mARU-Net network structure was established by embedding the CBAM attention module in the residual block. Finally, the model was normalized using the latest group normalization.

Following this section, the theoretical background is introduced in Section 3.1. The simplification and optimization of the U-Net structure is established in Section 3.2. CBAM-based feature extractions are introduced in Section 3.3.

### 3.1. Theoretical Background

Semantic segmentation is one of the three fundamental tasks of computer vision, which refers to the process of assigning a category label to each pixel in an image. Specifically, given an input image, the output of a semantic segmentation network is usually a feature map of the same size, and the feature vector of each pixel on the feature map represents the classification result at the corresponding location in the image. In contrast to image classification tasks, semantic segmentation networks not only need to understand the global information of the image, but also need to extract local information. Therefore, the overall design idea of semantic segmentation networks is to merge local and global features. Based on this, two fusion strategies are proposed, i.e., addition-based fusion and cascade-based fusion.

The U-Net is one of the most representative networks using a cascade-based fusion strategy. Compared with other networks that rely on large-scale datasets, U-Net requires only a few dozen images for training to generate segmentation results that are close to the ground truth. Based on global and local features of an image, the close connection between the encoding process and the decoding process in U-Net forms a unique U-shaped structure, which is suitable for the fine segmentation of images.

However, for fiducial mark segmentation tasks in this paper, the following drawbacks occur if we directly use the original U-Net:

1. The information carried by fiducial mark images with complex backgrounds is less than medical images. Consequently, it is necessary to adjust the network scale;
2. The output size is smaller than the input size in U-Net, so it is necessary to contract down-sampling operations and expand up-sampling operations in the path;
3. The U-Net does not use some new computing units to improve the performance of neural networks, such as residual blocks, normalization and modules.

As a result, and considering these shortcomings, this paper improves the original U-Net to adapt a specific fiducial mark segmentation task.

### 3.2. Segmentation Network Based on the U-Net

With regard to our fiducial mark segmentation task, directly using the original U-Net will result in overfitting, which reduces the model’s generalization ability. This is caused by excessive numbers of layers and parameters. Therefore, it is necessary to first simplify the U-Net based on the image features of fiducial marks by gradually reducing the number of convolution kernels during the training process. It is optimal to utilize the U-Net with the least number of convolution kernels as little loss of training accuracy as possible.

**Figure 1:**
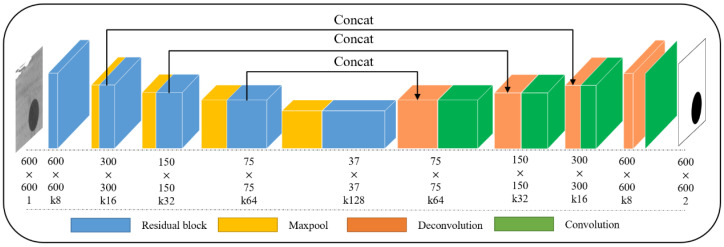
The architecture of the mARU-Net.

**Figure 2:**
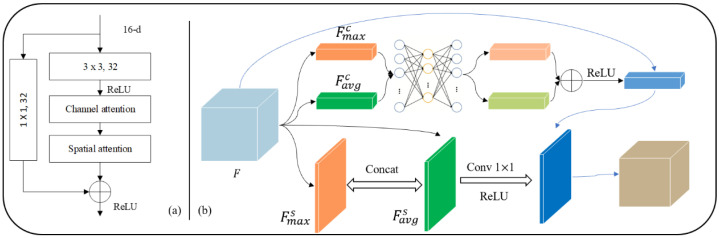
Additional operation units: (**a**) residual block and (**b**) convolutional block attention modules.

As shown in Figure 1, the network can be divided into two parts according to encoder and decoder processes: one encoder part is used for feature extraction, and the other decoder part for transposing convolution. In the left encoding path, the convolutional blocks of traditional U-Net are replaced by residual blocks, where the residual block is shown in Figure 2a. The single channel grayscale image including the marker point is used as the input, and after the first pooling by Maxpool, input into the residual block Res1. The final output is a feature matrix of 300 × 300 size and depth of 16. The entire down sampling encoding process was scaled down four times through pooling operations. In the right decoding path, we used deconvolution for up sampling, setting an up-sampling layer with a step size of 1 and a convolution kernel size of 3. The size of the feature vector is doubled, and the depth of the feature layer is reduced layer by layer. After each fusion, feature information with the same scale is fused using convolutional layers, normalization layers, and ReLU layers. After repeating this up sampling operation three times in sequence, the final feature vector of 300 × 300 × 16 is obtained.

The residual block can significantly improve training speed and feature extraction ability without additional parameters and calculations in the original network [39]. Therefore, as shown in Figure 2a, an mRU-Net architecture is proposed by introducing residual blocks in the encoding process of the mU-Net. Specifically, such a residual block doubles the number of channels of the input feature map while retaining its spatial scale. Notably, jump connections between input and output feature maps prevent model degradation and help the objective function converge quickly.

Given a fiducial mark image with a complex background, it is first processed to a single-channel grayscale image which is taken as an input by the mRU-Net. In the encoding stage, four down sampling processes are performed to triple the number of channels of the input feature map while reducing its spatial size to half. In the decoding stage, there are also four up-sampling blocks. Each consists of a transposed convolutional layer and a convolutional layer. Before the last up-sampling block, the feature map is produced by transposed convolution in series with the corresponding feature map in the down sampling stage.

The mRU-Net combines low- and high-resolution information to more accurately locate pixels. This allows the system to avoid the difficulty of balancing the spatial information of the image and image segmentation details in the FCN, and improves image-segmentation accuracy of the fiducial mark.

### 3.3. CBAM-Based Feature Extractions from Specific Regions

The convolutional block attention module (CBAM) [40] is used in spatial and channel dimensions. It is designed to enhance the feature extraction of specific regions of fiducial mark images. Therefore, as shown in Figure 2b, the CBAM is embedded into the proposed mRU-Net to improve the ability of the segmentation model, which extracts features from specific regions of images without additional parameters and computing resources.

Two sampling results are inputted to a shared multilayer perception (MLP) for segmentation training. In the end, the channel attention module is generated through an additive and nonlinear fusion strategy.

The channel attention module $M_{c}\left(F\right)$ is formulated as follows:

(1) $$M_{c}\left(F\right)=\sigma (MLP(AvgPool\left(F\right)+MLP(MaxPool(F)))\;=\sigma (W_{1}\left(W_{0}\left(F_{avg}^{c}+F_{max}^{c}\right)\right))$$

where F represents the input feature map, and $F_{avg}^{c}$ and $F_{max}^{c}$ are the feature maps after global average pooling and global max pooling, respectively. σ is the sigmold activation function, and $W_{0}$ and $W_{1}$ are multilayer perceptron model parameters.

Based on $F_{avg}^{c}$ and $F_{max}^{c}$, a convolutional layer was designed to generate the optimal fusion method for these two feature matrices. The spatial attention region extraction model solved the problem of the “position” of fiducial mark images.

The spatial attention module $M_{s}\left(F\right)$ is formulated as follows:

(2) $$M_{s}\left(F\right)=\sigma (f^{n\times n}([AvgPool\left(F\right);MaxPool(F)]))=\sigma (f^{n\times n}(\left[F_{avg}^{s};\;F_{max}^{s}\right]))$$

where F represents the input feature map; $F_{avg}^{s}$ and $F_{max}^{s}\;$ are feature maps after global average pooling and max pooling along channel dimension, respectively; σ is the sigmold activation function; the spatial attention module performs better here when n=7.

Many research studies have indicated that the classification effect is better if the channel attention module is ahead of the spatial attention module. Therefore, the two CBAM attention modules are embedded into each residual block in the U-Net encoding process, as shown in Figure 2. Finally, the mARU-Net is established, where A represents CBAM, and R stands for the residual block.

## 4. Experiments and Performance

### 4.1. Dataset Description and Metrics

The dataset used in our experiments contained a limited number of fiducial marker images with complex backgrounds. This article only obtained 33 original images in industrial production and an additional 7 synthetic images with extreme background complexity. Then, the Augmentor was used to enhance the synchronized data of the original image and label, increasing the number of images from 40 to 600. The production process of the custom dataset for fiducial mark inspection included segmentation annotation generation, detection annotation generation, data augmentation, and dataset division.

In order to maintain the statistical reliability of the results, ten people were selected from professional and non-professional groups as markers to generate segmentation and detection annotations. Specifically, the edge of a fiducial mark was identified and drawn by five people randomly selected from the ten individuals mentioned above, and the one closest to the edge of the original fiducial mark was selected by the other five persons through voting, and was filled with binary values. Then, center coordinates $\left(x,y\right)$ and the radius r of a fiducial mark were measured by ten individuals separately, and the average values of x, y, and r were calculated separately to serve as a reference for detection. Lastly, we adopted data augmentation approaches to expand the number of samples by 15 times and divide the dataset into training and testing sets using a ratio of 7:3.

To further evaluate the performance of the proposed method, a quantitative analysis based on the following two indicators was undertaken:

(3) $$mIoU=\frac{TP}{TP+FP+FN}$$

(4) $$Dice=\frac{2*\left|X⋂Y\right|}{\left|X\right|+\left|Y\right|}$$

where TP represents the number of correctly segmented foreground pixels, FP denotes the number of falsely segmented foreground pixels, and FN is the number of falsely segmented background pixels. X and Y represent the foreground region of the predicted segmentation map and the ground-truth map. Both of these indicators were ranged $\left[0,1\right]$, the higher the value, the better the performance.

### 4.2. Ablation Study

We conducted an ablation study to verify the effects of each of the four components of our proposed approach. The four improved networks at different stages, mU-Net, mRU-Net, mAU-Net, and mRU-Net, were trained to compare improvements. The mU-Net is a simplified U-Net, and the mRU-Net replaces convolutional blocks with residual blocks based on the mU-Net. The mAU-Net embeds the CBAM into the convolution block based on the mU-Net, and the mARU-Net embeds the CBAM into the residual block based on the mRU-Net.

Hyperparameters are key to controlling the structure, function, and efficiency of a model. Before network training, the training hyperparameters are shown in Table 1. The experimental software and hardware platforms in this article are as follows: Hardware platform: Consumer grade GeForce MX150 (2048 MB) graphics card; Software platform: Microsoft Windows 10.0.17134, Python 3.6.2, PyTorch1.0.1+cuda101.

Segmentation accuracy and efficiency values of the four networks are shown in Table 2. Data comparisons showed that introducing the residual block and the CBAM into the network improved segmentation accuracy, and that the performance of the mARU-Net was the best.

Compared with the mU-Net, the mRU-Net improved accuracy by 1.755%, the mAU-Net increased accuracy by 1.621%, and the mARUz-Net improved accuracy by 3.015%. The introduction of residual blocks and CBAM led to an increase in training time but real-time system efficiency was not affected.

**Figure 3:**
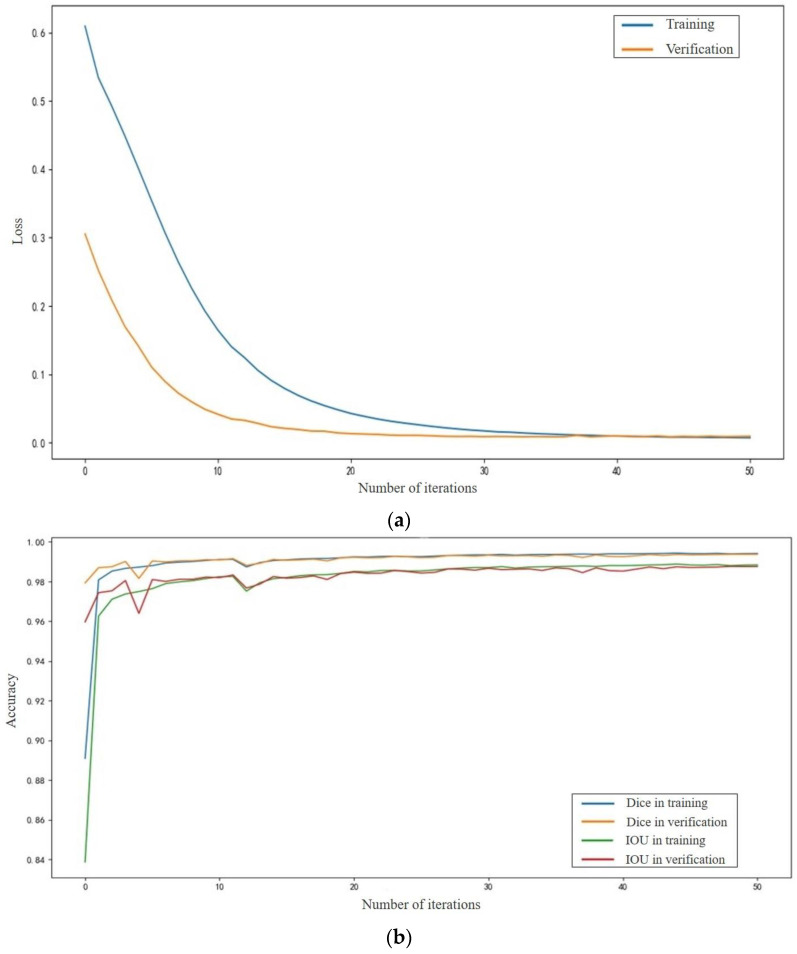
Loss and accuracy curves for the mARU-Net. (**a**) Loss curve; (**b**) Accuracy curve.

Loss and accuracy curves for the mARU-Net in training and verification are shown in Figure 3a,b, respectively.

### 4.3. Image Segmentation of Fiducial Marks

**Figure 4:**
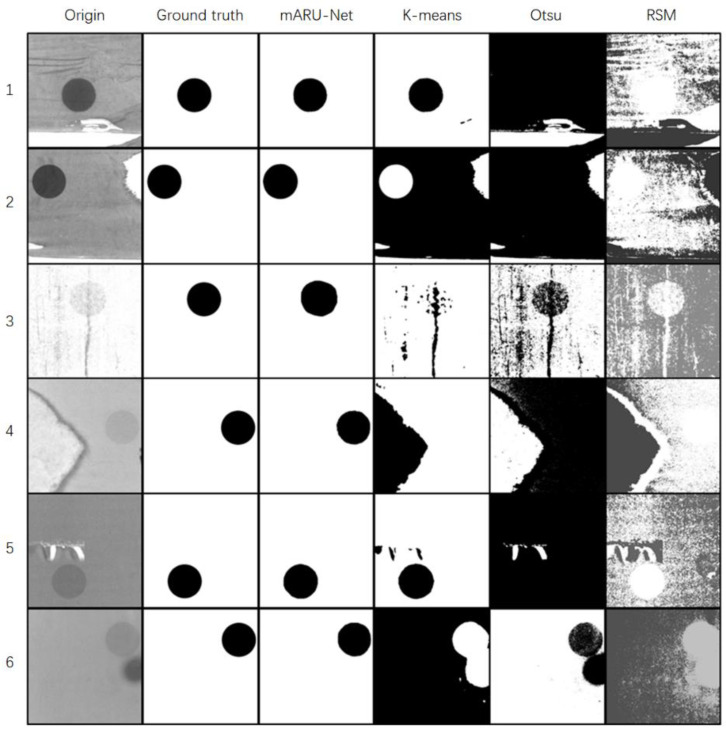
Segmentation results for fiducial mark samples with complex backgrounds using different methods.

Six fiducial mark images with complex backgrounds were taken as segmentation examples. Original images, ground truth, and segmentation results using the mARU-net, the K-means method, the Otsu method, and the region split and merge (RSM) method are shown in Figure 4.

Figure 4 shows that the K-means method and the Otsu method more or less under-segmented and over-segmented images, and that the RSM method did not clearly segment fiducial marks due to high background noise. The mARU-Net was not interfered with by textures, defects, noises, etc. In complex backgrounds, segmentation results were closer to the ground truth.

**Figure 5:**
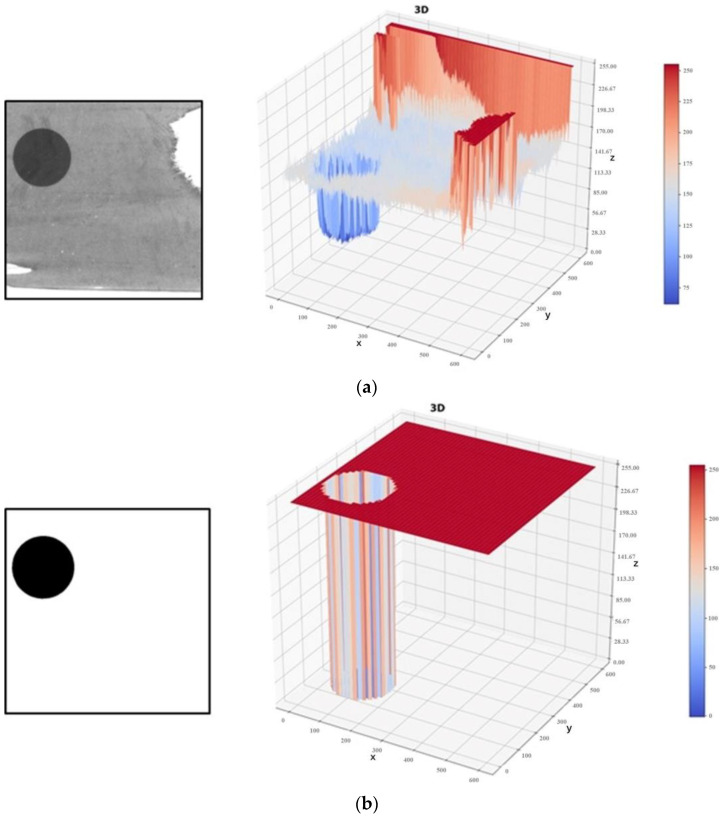
An image and gray levels before and after segmentation. (**a**) Original sample image and its gray-level distribution map; (**b**) Segmentation results using mARU-Net.

An original sample image, its gray-level distribution map, and segmentation results using the mARU-Net are shown in Figure 5a,b, respectively.

### 4.4. Detection of Fiducial Marks

**Figure 6:**
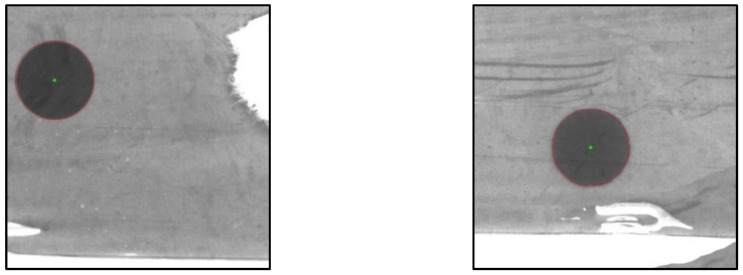
Two examples of fiducial mark detection.

In order to verify the segmentation accuracy of the mARU-Net, the centroid method was used to detect the center coordinates and radius of fiducial marks. Two detection examples are shown in Figure 6.

Based on camera calibration (11.26 μm/pixel), pixels at the center and radius of fiducial marks were converted into coordinates and length, and then compared with mean values obtained by manual labeling to calculate deviations, as shown in Table 3.

Table 3 shows that the maximum deviation of detection results of fiducial marks was less than 30 microns after image segmentation using mARU-Net. Therefore, mARU-Net met the accuracy requirements of fiducial mark image segmentation with complex backgrounds and helped improve PCB production efficiency.

## 5. Discussion

A mARU-Net was proposed to resolve the problem of fiducial mark segmentation in complex background images. Compared to the original U-Net structure, the segmentation accuracy Dice of mARU-Net was improved to 99.378%. The number of parameters and training times were at moderate levels. From the segmentation results of complex background images, it can be seen that the mARU-Net model proposed in this paper had better performances than algorithms such as OTSU, K-means, and CANNY. Circle detection efficiency was higher and deviations were smaller.

The mARU-Net method effectively segmented fiducial mark images with complex backgrounds. However, the method we proposed in this article cannot achieve good results for other low-quality fiducial mark images, such as edge reflection, size mixing, edge blurring, and other complex and unfavorable detection situations. For instance, there is reflection on the edges of fiducial mark images from actual detection processes. Edge reflections can cause white areas with sharply increased grayscale values at edges, resulting in incomplete fiducial mark areas. This method presents great challenges in segmenting such images.

In future work, we will focus on the problem of fiducial mark segmentation in edge reflective images which are interfered with by reflective regions. We will explore appropriate algorithms for the edge segmentation of fiducial marks using edge reflection interference. Furthermore, our next steps will be to explore the influence of different low-quality image conditions, such as size mixing, edge blurring, etc., on image segmentation and detection. We will also adopt appropriate methods to improve the accuracy of low-quality fiducial mark images.

## 6. Conclusions

This article focused on the problem of low recognition accuracy of PCB fiducial marks in complex backgrounds. We proposed the mARU-Net method in performing segmentation for images with complex backgrounds, followed by corresponding detection. The experimental results showed that the proposed method for detecting PCB fiducial marks had improved adaptability and accuracy.

Due to the complex background of PBC fiducial marks, there are significant challenges in the segmentation and detection of fiducial mark images. In this paper, the mARU-Net was proposed for the image segmentation of fiducial marks with complex backgrounds to improve detection accuracy. The mARU-Net was formed based on a simplified U-Net. In the encoding path, residual blocks were used to replace convolutional blocks in the traditional U-Net. To further improve image-segmentation performances of the network, the attention model CBAM was embedded into residual blocks.

Experimental studies showed that the effectiveness of the proposed network model was verified by comparing it with different improved network stages and typical segmentation algorithms using a customized fiducial mark image dataset. Based on segmentation results, the centroid method was used for circle detection, and the display accuracy of the results met detection accuracy requirements.

## Figures and Tables

**Table 1:** Hyperparameters.

Epochs	Lr	Optim	Loss	GN	Batchsize
51	0.0001	Adam	Dice Loss	2	1

**Table 2:** Segmentation accuracy and efficiency values of the four networks.

No.	Network Model	Number of Parameters	Training Time per Epoch(s)	Val Dice	Val IOU	Accuracy	Recall
1	mU-Net	538,391	169	0.964	0.957	0.978	**0.926**
2	mRU-Net	320,911	131	0.981	0.980	0.990	0.905
3	mAU-Net	541,503	218	0.980	0.974	0.987	0.910
**4**	**mARU-Net**	**324,023**	**179**	**0.994**	**0.988**	**0.994**	0.899

The best results are in bold.

**Table 3:** Detection results of the fiducial marks.

	x (μm)			y (μm)			r (μm)		
	GT	Detection	Deviation	GT	Detection	Deviation	GT	Detection	Deviation
1	1212.89	1216.08	−3.19	1908.60	1902.94	5.66	1042.57	1026.88	15.69
2	2985.44	2995.16	−9.72	3649.62	3636.98	12.64	1021.70	997.63	24.07
3	1878.16	1869.15	9.01	1622.37	1632.72	−10.35	1320.54	1345.41	−24.87
4	2650.89	2668.13	−17.24	2269.42	2257.33	12.09	997.57	1005.94	−8.37
5	1778.42	1798.78	−20.36	1834.63	1805.69	28.94	1226.38	1207.36	19.02
6	3036.65	3047.09	−10.44	1984.78	2007.34	−22.56	1612.05	1637.38	−25.33
7	2793.34	2787.65	5.69	1057.00	1042.14	14.86	1018.58	1004.37	14.21
8	2738.93	2714.85	24.08	2701.94	2706.28	−4.34	1645.06	1625.61	19.45

## Data Availability

Data are contained within the article.
